# *Klebsiella pneumoniae* Is Able to Trigger Epithelial-Mesenchymal Transition Process in Cultured Airway Epithelial Cells

**DOI:** 10.1371/journal.pone.0146365

**Published:** 2016-01-26

**Authors:** Laura Leone, Francesca Mazzetta, Daniela Martinelli, Sabatino Valente, Maurizio Alimandi, Salvatore Raffa, Iolanda Santino

**Affiliations:** 1 Department of Clinical and Molecular Medicine, Sapienza University of Rome, Rome, Italy; 2 Microbiology Unit, Azienda Ospedaliera Sant’Andrea, Rome, Italy; 3 Cellular Diagnostics Unit, Azienda Ospedaliera Sant’Andrea, Rome, Italy; Quuen's University Belfast, UNITED KINGDOM

## Abstract

The ability of some bacterial pathogens to activate Epithelial-Mesenchymal Transition normally is a consequence of the persistence of a local chronic inflammatory response or depends on a direct interaction of the pathogens with the host epithelial cells. In this study we monitored the abilities of the *K*. *pneumoniae* to activate the expression of genes related to EMT-like processes and the occurrence of phenotypic changes in airway epithelial cells during the early steps of cell infection. We describe changes in the production of intracellular reactive oxygen species and increased HIF-1α mRNA expression in cells exposed to *K*. *pneumoniae* infection. We also describe the upregulation of a set of transcription factors implicated in the EMT processes, such as Twist, Snail and ZEB, indicating that the morphological changes of epithelial cells already appreciable after few hours from the *K*. *pneumoniae* infection are tightly regulated by the activation of transcriptional pathways, driving epithelial cells to EMT. These effects appear to be effectively counteracted by resveratrol, an antioxidant that is able to exert a sustained scavenging of the intracellular ROS. This is the first report indicating that strains of *K*. *pneumoniae* may promote EMT-like programs through direct interaction with epithelial cells without the involvement of inflammatory cells.

## Introduction

The Epithelial-Mesenchymal Transition (EMT) is a physiological process that takes place in multicellular organisms, and it is characterized by dramatic changes of epithelial cells that loose their differentiated phenotype, to acquire ex novo mesenchymal features. This process requires rearrangements of the intercellular junctions, changes in apical-basal polarity and, sometimes culminates with the acquisition of motility and invasiveness, through the reorganization of the cytoskeleton dynamics [1].

In epithelial cells, the EMT program is switched on by many transcription factors (TFs), including Snail, Zeb, Slug and Twist. Working in tandem with multiple signaling pathways including TGF-β, Wnt, Notch and NF-κB (nuclear factor kappa-light-chain-enhancer of activated B cells), their activity is thought to regulate the expression of genes related to epithelial and mesenchymal phenotype and suppress the expression of E-cadherin [2]. A second pathway involved in the induction of EMT-like processes is sustained by hypoxia and cellular stress with subsequent generation of intracellular reactive oxygen species (ROS) [3,4]. This is an event which is frequent in the microenvironment of infected tissues, and triggers pathways through the induction of the hypoxia-inducible factor-1α (HIF-1α) leading to the activation of histone deacetylase (HDAC) 3, essential for the establishment of EMT-like processes and metastasis [5]. Once stabilized, HIF-1α translocates to the nucleus where directly induce the expression of Twist by binding to DNA regulatory sequences, called hypoxia response elements (HREs), localized in the Twist proximal promoter region [6].

Recent studies have documented that EMT is also involved in cancer development and progression, inflammatory and tissue repair processes and organ fibrosis [7,8]. In this case, the induction of the EMT process can be mediated and sustained by the interactions of bacterial pathogens with the epithelium. To this extent, peculiar is the ability of some entero-adherent bacteria to trigger and maintain a chronic inflammatory environment prerequisite for the subsequent possible development of EMT-like phenotypes in cells continuously exposed to inflammatory stress, or induced by the pathogen to activate specific transcriptional programs [9]. Alterations in signaling pathways during enteric infections by *Helicobacter pylori* and *Enterococcus faecalis* can cause intracellular stress with tissue/organ damage [10,11] and can also promote the acquisition of malignant phenotype [12–14].

This is also true for the respiratory tract, where the presence of chronic inflammation is thought to contribute to the genesis of neoplastic and non-neoplastic airway diseases, such as idiopathic pulmonary fibrosis (IPF) [15]. In this case, epithelial cells become chronically exposed to the Transforming Growth Factor (TGF)-β1 and so they initiate an EMT process, which is also sustained by the microenvironment and culminates in the acquisition of a myofibroblast-like phenotype [16,17].

Pseudomonas aeruginosa infection is a further example of the unbalanced homeostasis of the microenvironment that, during chronic infections, synergizes with the TGF-β1 to drive airway epithelial cells toward the transition to a mesenchymal-like phenotype [18].

*Klebsiella pneumoniae* (*Kp*) is a Gram-negative bacterium causing community-acquired and nosocomial infections, for its ability to colonize different tissues, including lungs, urinary tract, airways and skin wounds [19]. The intensive use of carbapenems for the treatment of severe infections from bacteria producing extended spectrum of β-lactamases (ESBLs) caused, in recent years, the appearance of carbapenem-resistant *Kp* strains worldwide [20–22].

We recently demonstrated that *Kp* strains have the ability to infect epithelial cells in many in vitro models, including colon, skin, lung and kidney epithelial cells, and that cell damages are more evident in cells infected by the carbapenems and colistin resistant strains, leading epithelial cell to an anticipated cell death [23].

In the present study we attempted to determine the abilities of different *Kp* strains to induce gene expression profiles and phenotypic changes in cultured epithelial cells possibly related to the activation of EMT-like programs and to identify mechanisms of induction and time frame of their occurrence. To this purpose, we selected the A549 airway epithelial cells that show typical characteristics of alveolar type II cells, but are also able to quickly modulate shape and morphology in course of biochemical stress.

To infect A549 cells, we used a set of *Kp* strains isolated from samples of hospitalized and ambulatory patients and differently resistant to carbapenems and colistin drugs, to study the production of intracellular reactive oxygen species (ROS) and the fluctuation of HIF-1α gene during the early phase of infection. Furthermore, in search of the biochemical pathways involved in the morphological changes of A549 cells occurring in the early steps of *K*. *pneumoniae* infection, we monitored the expression and identified variation of a set of transcription factors implicated in the EMT processes, such as Twist, Snail and ZEB [9].

We show the upmodulation of intracellular ROS levels and increased HIF-1α mRNA expression in cells exposed to *K*. *pneumoniae* infection. We also describe the activation of TFs implicated in the EMT processes, indicating that the phenotypical changes of A549 cells appreciable after few hours from the infection are regulated by the activation of transcriptional pathways, driving epithelial cells to EMT. These effects seem to be effectively counteracted by scavenging of intracellular ROS.

## Materials and Methods

### Bacterial strains

Four carbapenemase producing *Kp* strains (#UR1, #SP1, #SP2, SP3) belonging to the sequence type 512 clone (ST512) and carrying the blaKPC3 gene were isolated from urine or sputum samples [24] of hospitalized patients (Table 1) and cultured in accordance with guidelines approved by the management of the Sant’Andrea Hospital for routine care purposes. *Kp* strain #PS1 was isolated from pharyngeal swab of ambulatory patient and identified and characterized for drug susceptibility as above. For this study no formal ethical approval was needed because all the data were treated anonymously and the strains were remnants from patient samples collected for routine diagnosis. The anonymization of the samples was performed by the technical staff of the Microbiology Unit of the Sant’Andrea Hospital and the authors had neither interaction with the patients nor access to their personal data.

**Table 1:** Characteristics of *K*. *pneumoniae* strains [23].

Specimen code	Patients (yrs/sex)	Sample/anatomic site of isolation	Location unit	Strains	KPC	Sequence type
**#UR1**	77/F	Urine	Vascular surgery	*KPC-Kp*	3	512
**#SP1**	75/M	Sputum	Cardiac surgery	*KPC-Kp*	3	512
**#SP2**	68/F	Sputum	Infectious diseases	*KPC-Kp Col-R*	3	512
**#SP4**	66/F	Sputum	Emergency medicine	*KPC-Kp Col-R*	3	512
**#PS1**	32/M	Pharyngeal swab	Ambulatory	*Kp*		

Legend: *Kp*: *Klebsiella pneumoniae*; *KPC-Kp*: Carbapenem-resistant *K*. *pneumoniae*; *KPC-Kp Col-R*: *KPC-Kp* resistant also to colistin.

The bacterial identification and drug susceptibility of *Kp* isolates were tested using the Vitek 2 automated system (bioMérieux, Craponne, France). E. Coli ATCC 25922 was used as quality control strain. Susceptibility to colistin was also confirmed using the E-test (bioMérieux) according to the manufacturer’s instructions and interpreted using the EUCAST cut-off. Imipenem resistance was defined as an MIC of ≥8 μg/mL, and colistin resistance was defined as an MIC of ≥2 μg/mL. Phenotypic screening for carbapenemases was carried out on all isolates by using the modified Hodge test using disks of meropenem 10 μg and ertapenem 10 μg according to the manufacturer’s instructions. The molecular typing of *KP*C-type β-lactamase was identified by polymerase chain reaction and sequencing and the clonality of the isolates was investigated by multilocus sequence typing MLST as previously described [24].

Fresh bacteria previously grown overnight at 37°C in brain heart infusion broth (BD Diagnostic Systems, Franklin Lakes, NJ, USA) were used for the experiments.

### Cell cultures, treatments and bacterial infection

The human alveolar epithelial cells A549 [25] was cultured in Dulbecco’s modified Eagle medium (DMEM) supplemented 10% fetal bovine serum (FBS) and antibiotics. For the bacterial infection the cells were grown until approximately 80% of confluence. Before infection, cells were maintained overnight in basal medium without serum or antibiotics. Subsequently, cells were infected with the different strains of overnight-grown bacteria at a multiplicity of infection (MOI) ranging from 25 to 200:1 and centrifuged for 4 min at 200g at 25°C. Infected plates were then incubated for 2h at 37°C/5% CO2 in humidified incubator, washed three times with PBS to remove unbound bacteria and then incubated again for 2h at 37°C. For treatments with growth factors, cells were serum starved for 12h and then incubated with 50 ng/mL TGF-β1 (PeproTech Inc., Rocky Hill, NY, USA) for 4h. For treatments with ROS scavenger, A549 cells were pretreated with 3,5,4’-trihydroxy-trans-stilbene (resveratrol, 50 μM; Sigma–Aldrich, St. Louis, MO, USA) left in the basal medium without serum or antibiotics for 12h before the infection.

### Morphological analysis

The cell cultures were observed on an Axiovert 200 inverted microscope (Zeiss, Oberkochen, Germany) equipped with differential interference contrast (DIC) optics and with Axiovision image analysis system (Zeiss).

### Immunofluorescence

A549 uninfected and infected cells were fixed with 4% paraformaldehyde followed by treatment with 0.1M glycine for 20 min at 25°C and with 0.1% Triton X-100 for additional 5 min at 25°C to allow permeabilization. Cells were then incubated with the following primary antibodies: anti-CD326/EpCAM PE (1:10 in PBS; Miltenyi Biotec, Bergish Gladbach, Germany), anti-vimentin (1:50 in PBS; Dako, Glostrup, Denmark) and anti-HIF-1α (1:100 in PBS, Novus Biologicals, Littleton, CO, USA). The unconjugated primary antibodies were visualized, after appropriate washing with PBS, by using goat anti-mouse IgG–FITC (1:50 in PBS; Cappel Research Products, Santa Ana, CA, USA) for 30 min at 25°C. Nuclei were stained with DAPI (1:10000; Sigma Chemicals, St Louis, MO, USA). Coverslips were finally mounted with Mowiol in PBS for observation. Fluorescence signals were analyzed by ApoTome System (Zeiss) connected with an Axiovert 200 inverted microscope (Zeiss). The percentage of CD326- or vimentin- positive cells was analyzed counting at least 500 cells, observed, for each experimental point, in ten microscopic fields randomly taken from three different experiments. Quantitative analysis of the HIF-1α fluorescence intensity for cytoplasmic area was performed by the analysis of at least 100 cells for each sample in 5 different fields randomly taken from three independent experiments and performed by the Axiovision software (Zeiss)

### Cell viability assays

The analysis of cell viability was determined by flow cytometry. A cell suspension of uninfected or *K*. *pneumoniae*-infected A549 cells was stained with 1 μg/mL of Propidium Iodide Solution (Miltenyi Biotec GmbH, Bergisch Gladbach, Germany) and analyzed by MACSQuant Analyzer flow cytometer (Miltenyi Biotec GmbH). Excitation and emission wavelengths were 488 and 585 nm respectively (B2 channel). The red fluorescence signal was analyzed by MACSQuantify software (Miltenyi Biotec GmbH) from three independent experiments with evaluation of at least 20,000 events for assay.

### Reactive oxygen species detection

For ROS detection the uninfected or *K*. *pneumoniae*-infected A549 cells were incubated with 2’,7’-dichlorofluorescein diacetate (DCFH-DA, Sigma-Aldrich, St. Louis, MO, USA) 5 μM for 10 min at 37°C, extensively washed with PBS, trypsinized, pelleted, resuspended in pre-warmed medium and collected with MACSQuantH Analyzer flow cytometer (Miltenyi Biotec GmbH). The excitation and emission wavelengths were 488 and 525 nm respectively (B1 channel). The green fluorescence signal was analyzed by MACSQuantify software (Miltenyi Biotec GmbH) and visualized on a three-decade log scale. The mean fluorescence intensity (MFI±SE) was calculated from three independent experiments with evaluation of at least 20,000 events for assay [26].

The generation of intracellular ROS was confirmed by confocal quantitative analysis [27]. The A549 cells, treated as above, were immediately observed under an Axioskop 2 microscope equipped with Pascal LSM 5 confocal laser scan (Zeiss) using an argon laser with a 488 nm excitation band. The emission long pass was a 505 filter: laser intensity, pinhole diameter and photomultiplier settings were kept constant for every experiment. The fluorescence intensity (FIU, Fluorescence Intensity Units) was measured by Axiovision software (Zeiss) evaluating at least 300 cells for each condition in three different microscopic fields. To visualize the differences of intensity and intracellular localization of DCFH-DA signal, digital images were further analyzed through a glow-scale of pseudo-color [28]. This function of the Zeiss software expresses the fluorescence intensity in a pseudo-color scale, in which white is the highest and brown-red is the lowest intensity value in gray scale levels.

### Primers

Oligonucleotide primers for target genes and for the housekeeping gene (GAPDH) were chosen with the assistance of the Oligo 5.0 computer program (National Biosciences, Plymouth, MN, USA) and purchased from Invitrogen (Carlsbad, CA, USA). The primers used are listed in Table 2. For each primer pair, we performed no-template control and no-reverse-transcriptase control (RT negative) assays, which produced negligible signals.

**Table 2:** Primers used for target and housekeeping genes.

Gene	Primer sequences	Efficiency	r^2^	Slope
**GAPDH**	For 5’-CATCAGCAATGCCTCCTGCAC- 3’	100%	0.999	-3.39
	Rev 5’-GTCATGAGTCCTTCCACGATACCAA- 3’			
**HIF-1α**	For 5’-GATAGCAAGACTTTCCTCAGTCG- 3’	98%	0.997	-3.1
	Rev 5’-TGGCTCATATCCCATCAATTC- 3’			
**SOD-1**	For 5’ -GGCCAAAGGATGAAGAGAGGCATGTT- 3’	95%	0.996	-3.04
	Rev 5’—GACCACCAGTGTGCGGCCAA- 3’			
**SOD-2**	For 5’ -GGTGGAGAACCCAAAGGGGAGTTG- 3’	96%	0.997	-3.08
	Rev 5’ -TTATTGAAACCAAGCCAACCCCAACCT- 3’			
**Catalase**	For 5’ -GGAGCAGGGGCCTTTGGCTACTT- 3’	94%	0.992	-3.01
	Rev 5’ -TCCAGCAACAGTGGAGAACCGAACT- 3’			
**ZEB 1**	For 5’-GGGAGGAGCAGTGAAAGAGA- 3’	95%	0.999	-3.4
	Rev 5’-TTTCTTGCCCTTCCTTTCTG- 3’			
**ZEB 2**	For 5’-AAGCCAGGGACAGATCAGC- 3’	110%	0.997	-3.01
	Rev 5’-CCACACTCTGTGCATTTGAACT- 3’			
**Snail 1**	For 5’-GCTGCAGGACTCTAATCCAGA- 3’	100%	0.998	-2.8
	Rev 5’-ATCTCCGGAGGTGGGATG- 3’			
**Snail 2**	For 5’-TGGTTGCTTCAAGGACACAT- 3’	99.3%	0.996	-3.34
	Rev 5’-GCAAATGCTCTGTTGCAGTG- 3’			
**Twist 1**	For 5’-AGCTACGCCTTCTGGTCT- 3’	96.4%	0.998	-3.4
	Rev 5’-CCTTCTCTGGAAACAATGACATC- 3’			
**Twist 2**	For 5’-CATGTCCGCCTCCCACTA- 3’	105%	0.999	-3.4
	Rev 5’-GCATCATTCAGAATCTCCTCCT- 3’			
**ITGB4**	For 5’ -GGGAAAAAGCAAGACCACACC- 3’	101%	0.997	-3.2
	Rev 5’ -CCCTCTGTTCCACCTGCTTC- 3’			
**E-Cad**	For 5’ -TGGAGGAATTCTTGCTTTGC- 3’	99%	0.998	-3.15
	Rev 5’ -CGCTCTCCTCCGAAGAAAC- 3’			

### RNA extraction and cDNA synthesis

RNA was extracted using the TRIzol method (Invitrogen) according to manufacturer’s instructions and eluted with 0.1% diethylpyrocarbonate (DEPC)-treated water.

Total RNA concentration was quantitated by spectrophotometry and the quality was assessed by measuring the optical density ratio at 260/280 nm. RNA samples were stored at -80°C. After denaturation in DEPC-treated water at 70°C for 10 min, 1 μg of total RNA was used to reverse transcription using iScript™ cDNA synthesis kit (Bio-Rad, Hercules, CA, USA) according to manufacturer’s instructions.

### PCR amplification and real-time quantitation

Real-time RT-PCR was performed using the iCycler Real-Time Detection System (iQ5 Bio-Rad) with optimized PCR conditions. The reactions were carried out in 96-well plate using iQ SYBR Green Supermix (Bio-Rad) adding forward and reverse primers for each gene and 1 μl of diluted template cDNA to a final reaction volume of 15 μl. All the assays included positive and negative controls were replicated three times. The thermal cycling program was performed as follows: an initial denaturation step at 95°C for 3 min, followed by 45 cycles at 95°C for 10 sec. and 60°C for 30 sec.

Real-time quantitation was performed with the help of the iCycler IQ optical system software version 3.0a (Bio-Rad), according to the manufacturer’s guidelines. The relative expression of the GAPDH housekeeping gene was used for standardizing the reaction. The comparative threshold cycle (Ct) method was applied to calculate the fold changes of expression compared to control cells. Results are reported as mean ± standard deviation (SD) from three different experiments conducted in triplicates.

### Statistical Methods

Student’s T test was used to evaluate statistical significance among variables that assume normal distribution. The values are expressed as mean ± SD from three independent experiments. Mann-Whitney non-parametric test was used to compare variables that do not assume Gaussian distribution,. The values are expressed as median ± Interquartile Ranges (IR) from three independent experiments. The data are represented with the Tukey box-and-whisker plot; the central box represents the values from the lower to upper quartile (25th to 75th percentile), the middle line represents the median, and the horizontal lines represent the minimum and the maximum value of observation range. The one-way ANOVA test was used to compare three or more sets of unpaired data. To verify the existence of a linear trend between the group means one-way ANOVA post-test for trend was performed. The Dunnett's test was performed to compare the set of control data to the all other sets of experimental data.

The correlation measures were evaluated by the Pearson test (r) and by linear analysis of regression curve. *P* values <0.05 were assumed as statistically significant.

## Results

### The infection of *K*. *pneumoniae* induces early morphological changes in A549 cells that assume an EMT-like phenotype

**Fig 1:**
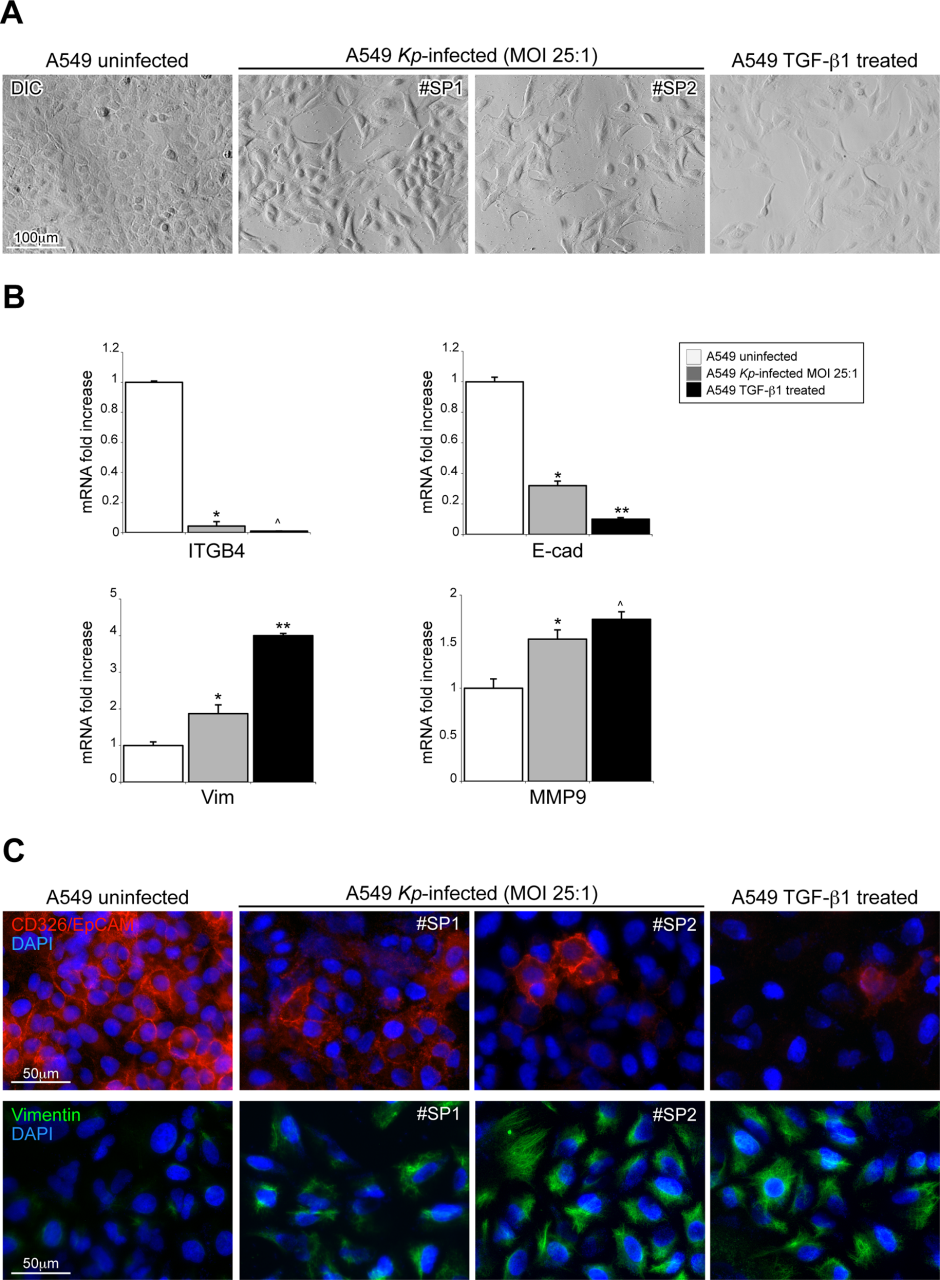
Analysis of early EMT-like phenotypic changes activation in the EMT processes in A549 cells infected with *K*. *pneumoniae* strains. (A) The morphologycal analysis by differential interference contrast microscopy of the *K*. *pneumoniae* infected cells shows an elongated morphology with reduction of intercellular contacts compatible with EMT-like phenotype, similar to that displays by monolayers exposed to TGF-β1. Conversely, uninfected A549 cells retain a typical epithelial morphology, with clear cell-cell adhesions. (B) The real time RT-PCR analysis performed in A549 *Kp*-infected cells at MOI 1:25 showed a downregulation of the genes encoding for epithelial adhesion molecules (ITGB4 and E-cad) and an upregulation of genes encoding for mesenchymal markers vimentin and MMP9. A459 TGF-β1-treated cells were used as positive control of EMT-induction. The mRNA levels were normalized respect to uninfected cells and results are expressed as mean ± SD. (Mann-Whitney test: *p<0.01 vs uninfected cells; **p<0.01 vs uninfected and *Kp*-infected cells, ^*p*<0.01 vs uninfected cells and not significant vs *Kp*-infected cells). (C) The immunofluorescence analysis reveals a reduction of CD326/EpCAM positive cells in *K*. *pneumoniae* infected cells as compared to uninfected cells with typical bordered staining (61% vs 94%: Mann-Whitney test: *p*<0.05 vs uninfected cells). Furthermore, the infected cells, in analogy of what observed in TGF-β1-treated cells, showed positivity rates of vimentin close to 100% respect to 31% of uninfected cells (Mann-Whitney test: *p*<0.05).

The early phases of *K*. *pneumoniae* infection are characterized by the appearance of morphological changes in infected cells that become visible already after few hours from the initial contact of bacteria with the epithelial cells. In particular, we noticed that A549 cells in contact with any of the K. pneumonia strains at a MOI ranging to 25–200:1, rapidly undergo to a rearrangements of cell shape, compatible for type of morphology and time-frame of occurrence, with the activation of pathways that normally regulate the acquisition of EMT-like phenotypes. Starting from this initial observation we wanted to deeply characterize the newly acquired morphology of A549 infected cells, monitoring cell-shape variations with a phase-contrast microscopy analysis. The A549 cells infected with any of the *Kp* strains showed an elongated morphology with reduction of intercellular contacts already after three to five hours from infection. This newly acquired morphology, suggestive of cells in epithelial-mesenchymal transition state was similar to that displayed by cells exposed to TGF-β1, meanwhile uninfected A549 cells had a typical epithelial morphology, with intact cell-cell adhesion structures (Fig 1A).

To further characterize the molecular culprits of these changes we looked at the potential variation in the expression of the typical adhesion molecules of epithelial cells, and at the activation of genes that promote mesenchymal properties in the infected monolayers. In particular, we monitored the mRNA expression of integrin beta 4 (ITGB4), Cadherin E (E-cad), vimentin and Metalloprotease-9 (MMP9) in A549 infected cells. The real time RT-PCR analysis performed in A549 infected cells showed a downregulation of the genes encoding for epithelial adhesion molecules ITGB4 and E-cad and an upregulation of genes encoding for vimentin and MMP9. These data are comparable to those obtained in the A549 treated with TGF-β1 (Fig 1B).

In addition, at the same experimental conditions we wanted to evaluate expression and organization of the transmembrane glycoprotein CD326/EpCAM and the vimentin intermediate filaments, by immunofluorescence. As expected, the quantitative analysis reveals the presence of a typical bordered CD326/EpCAM staining on the membrane of the majority of uninfected cells (Fig 1C). By contrast, its expression in A549 cells infected by *Kp* was less evident, with a reduction of CD326/EpCAM positive cells to 62%, as compared to uninfected cells (94%, IR 88–96%; *p*<0.05). Furthermore, in analogy of what observed in A549 cells treated with TGF-β1, all the infected cells showed positivity rates of vimentin close to 100% respect to 31% of uninfected cells (*p*<0.05; Fig 1C).

Therefore, we described in A549 cells that the ability of *K*. *pneumoniae* to modulate the expression of adhesion molecules typical of an EMT with a down-regulation of epithelial markers and to the up-modulation of mesenchymal markers. The effects induced in A549 cells by *K*. *pneumoniae* are similar to those generated by TGF-β1 treatment, a pivotal inductor of the EMT process.

### *K*. *pneumoniae* induces oxidative injury and cytotoxic effects in A549 epithelial cells

We previously demonstrated that *Kp* strains, are able to infect in vitro several types of tissue-specific epithelial cells [23]. Similarly, Cano et al. demonstrated that *K*. *pneumoniae* induces a cytotoxic effects in A549 cells at MOI ≥500:1 [29]. To evaluate the capacity of *K*. *pneumoniae* to induce cell damage also in our cellular model, we infected A549 cells with all the *Kp* strains at a MOI ranging to 25–200:1, to monitor the cell viability using Propidium Iodide (PI) in flow cytometric analysis.

**Fig 2:**
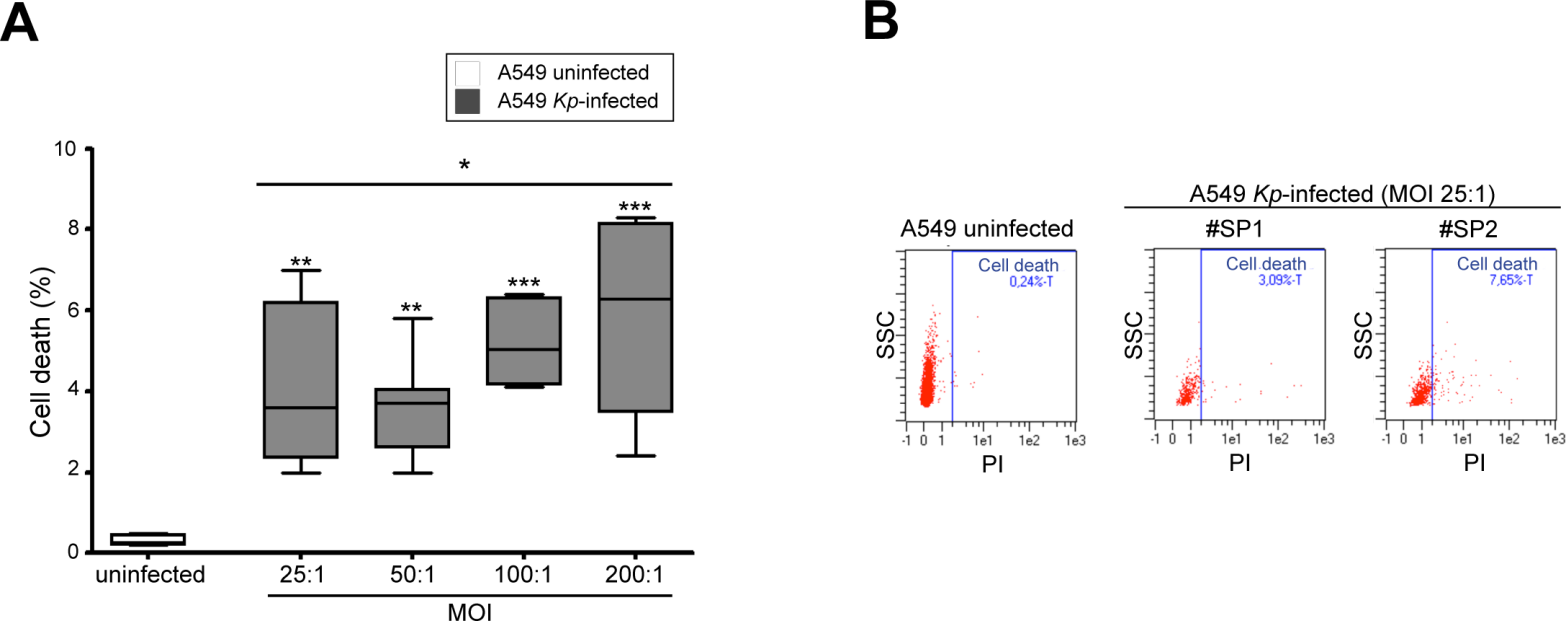
Analysis of cytotoxic effects induced by *K*. *pneumoniae* infection in A549 cells. (A) The cell viability assay by cytofluorimetric analysis of PI fluorescent signal shows that *K*. *pneumoniae* infection induces cellular damage mostly related to MOI (ANOVA post test for linear trend: *p<0.01; Dunnett’s test: **p>0.01 or ***p>0.001 vs uninfected cells). (B) The representative flow cytometry plots at MOI 25:1, at which we have observed the lowest cytotoxic effect, display an increased amount of cellular events in the right gate (necrotic cells) for the A549 infected by K.pneumoniae strains. Legend: PI: Propidium Iodide.

The FACS analysis shows that *K*. *pneumoniae* infection induces cell damages mostly related to MOI; in fact, the percentage of cell death was higher at MOI 100–200:1 in A549 infected by all the *K*. *pneumoniae* strains (*p*<0.0001; Fig 2A).

**Fig 3:**
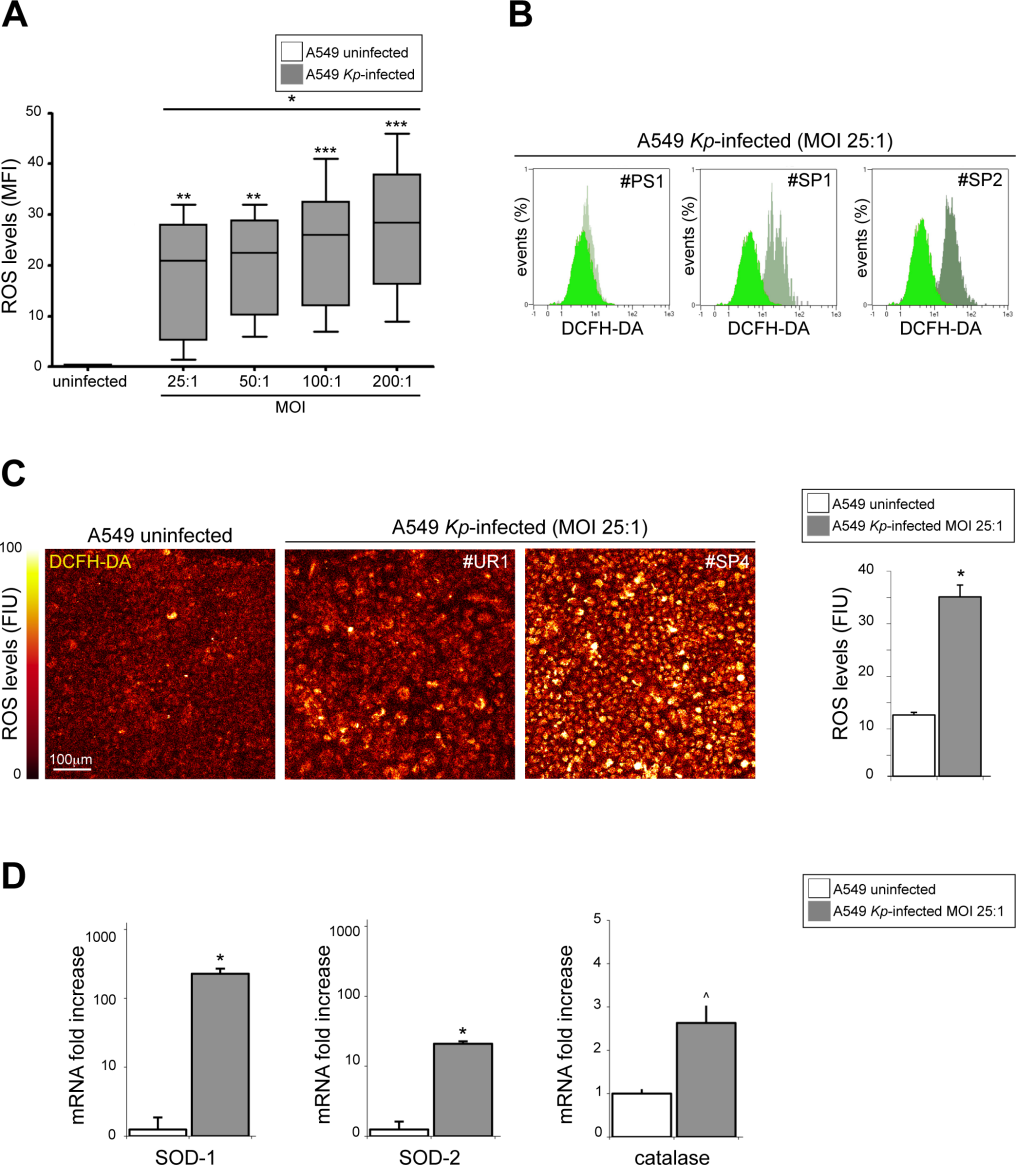
Evaluation of the intracellular ROS generation and the expression of antioxidant genes due to *K*. *pneumoniae* infection of A549 cells at lower MOI. (A) The assessment of intracellular ROS generation by cytofluorimetric analysis shows the modulation of intracellular ROS induced by *K*. *pneumoniae* infection. The ROS overproduction is related to MOI (ANOVA post test for linear trend: *p<0.05; Dunnett’s test: **p>0.05 or ***p>0.01 vs uninfected cells). The representative flow cytometry plots at MOI 25:1 show an enhancement of DCFH-DA signal in A549 *Kp*-infected (flat green) respect to uninfected cells (brilliant green). (B) The confocal quantitative analysis of intracellular DCFH-DA fluorescent signal confirms the overproduction of ROS species in A549 *Kp*-infected cells (Student T test: *p<0.0001). In the representative digital images, the differences in the levels of DCFH-DA signal were expresses in a pseudo-color scale of fluorescence intensity, in which white is the highest and brown-red is the lowest intensity value of gray scale levels. Legend: MFI: Mean Fluorescence Intensity; FIU: Fluorescence Intensity Units. (C) SOD-1, SOD-2 and catalase mRNA levels were evaluated by real time RT-PCR in *Kp*-infected cells at MOI 1:25 and normalized respect to uninfected A549 cells. Results are expressed as mean ± SD. (Mann-Whitney test: *p<0.01 or ^*p* = not significant vs A549 uninfected cells).

Since many bacterial species are able to induce oxidative stress in different epithelial tissues [10,11], we hypothesized that the cytotoxic effect exerted on A549 cells by higher MOI of *K*. *pneumoniae* would be related to the intracellular overproduction of ROS. To analyze the modulation of ROS production caused by *K*. *pneumoniae* infection, we infected A549 cells with various *Kp* strains at different MOI. The assessment of ROS generation by cytofluorimetric analysis shows that the oxidative stress induced by *K*. *pneumoniae* was related to MOI (*p*<0.05; Fig 3A).

Taken together, these data indicate that any of the *Kp* strains is able to induce a MOI-related cell death on A549 cells; moreover, the percentages of cytotoxicity registered in our test indicate the existence of a strong positive relation with the levels of intracellular reactive oxygen species induced by *K*. *pneumoniae* infection (r: +0.89, f: *p*<0.001), supporting the crucial role of oxidative stress in the cell death mechanisms observed.

### *K*. *pneumoniae* at lower MOI induces upmodulation of ROS intracellular levels and positive regulation of HIF-1α gene

To assess whether the ROS generated in course of *K*. *pneumoniae* infection would be able to upregulate the HIF-1α gene expression in A549 cells, we have exposed cultured cells grown in monolayers at MOI 25:1, the same conditions in which we observed the lowest cytotoxic effect (Fig 2B). The experimental conditions for the EMT induction were controlled treating A549 cells with TGF-β1 50ng/ml.

At the selected MOI, the intracellular ROS amounts were obviously higher in infected as compared to uninfected A549 cells (Fig 3B). Moreover, these findings were corroborated by confocal quantitative analysis of DCFH-DA fluorescent signal that confirms an intracellular overproduction of ROS for all A549 *Kp*-infected monolayers (*p*<0.0001; Fig 3C).

The stimulation of the oxidative stress in course of *K*. *pneumoniae* infection was also confirmed by the activation of counteracting pathways culminating with the upregulation of antioxidant genes [30]. We monitored the induction of the antioxidant enzymes Cu,Zn-superoxide dismutase (SOD-1) and Mn-superoxide dismutase (SOD-2) genes, in the infected monolayers and we found a significant variation of mRNA synthesis by real time RT-PCR (*p*<0.05). The results illustrated in Fig 3D, indicate that the SOD-1 and SOD-2 are synthesized to counteract oxidative stress, although with no appreciable differences in expression among the infective strains of pathogen. We also quantified the mRNA levels of the catalase gene, without identifying significant changes of its expression (Fig 3D).

**Fig 4:**
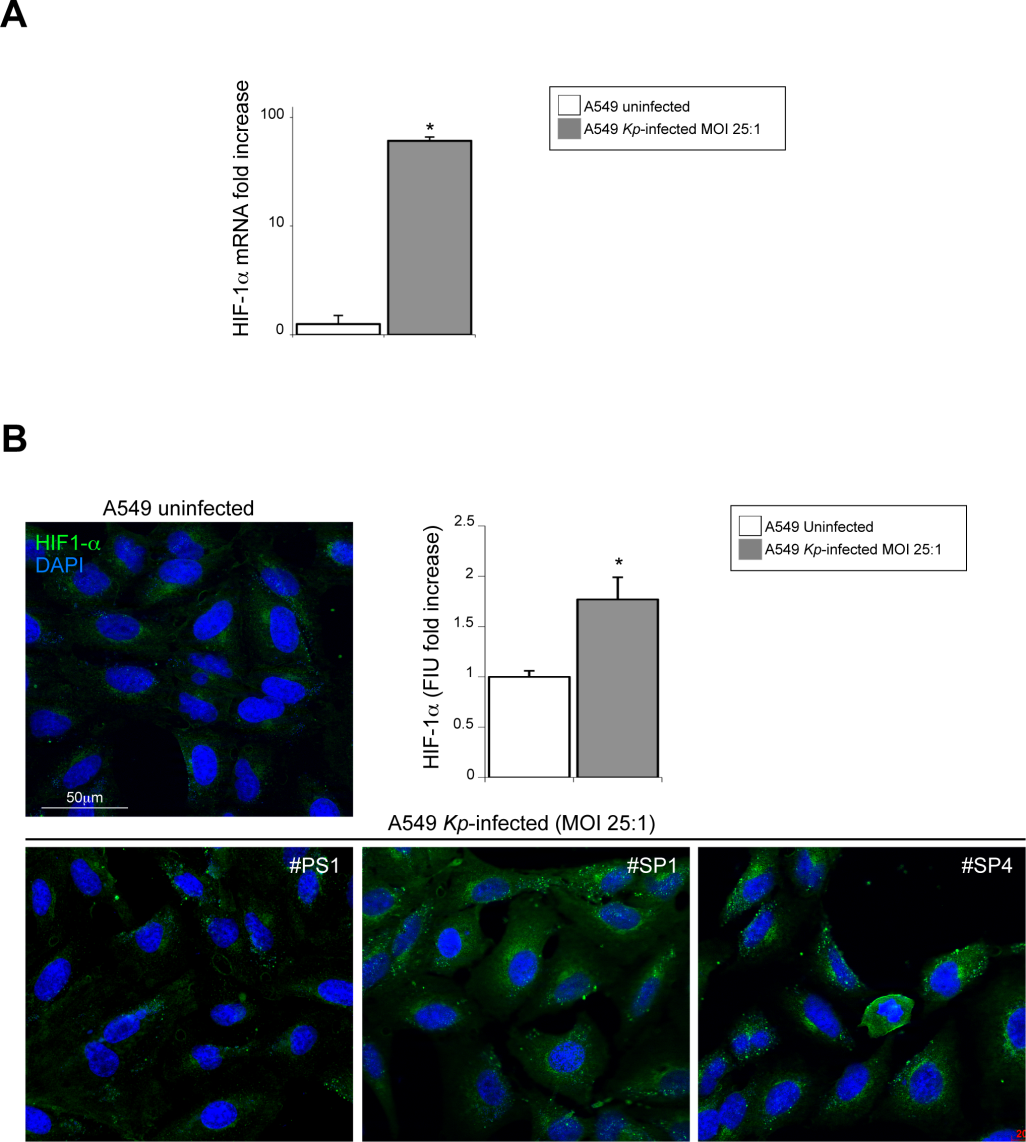
Evaluation of HIF-1α expression due to *K*. *pneumoniae* infection of A549 cells at lower MOI. (A) HIF-1α mRNA levels were evaluated by real-time RT-PCR in A549 cells infected with *K*. *pneumoniae* strains at MOI 1:25 and normalized respect to uninfected A549 cells. Results are expressed as mean ± SD. To observe a significant increase of HIF-1α mRNA expression in A549 infected monolayers (Mann-Withney test: *p<0.01 vs uninfected cells). (B) Quantitative immunofluorescence analysis shows a significant increase of the cytoplasmic HIF-1α signals in A549 exposed to the infection of the *K*. *pneumoniae* strains (Student T test: *p<0.001 vs uninfected cells). The fluorescence intensity for cytoplasmic area was performed by the analysis of at least 100 cells for each sample in 5 different fields randomly taken from three independent experiments. The results are expressed as mean ± CI 95%.

Finally, we looked at HIF-1α expression in the early steps of *K*. *pneumoniae* infection. HIF-1α is a master regulator of anaerobic metabolism and, during exposure to hypoxia and cellular stress, it undergoes redox regulation. It was not surprising to observe a significant increase of HIF-1α mRNA expression in A549 infected monolayers (*p*<0.01; Fig 4A). Similarly, quantitative immunofluorescence analysis shows a significant increase of the cytoplasmic HIF-1α signals in A549 exposed to the infection of the *Kp* strains (*p*<0.001; Fig 4B).

The amount of intracellular ROS are positively related to the levels of HIF-1α either detected by mRNA expression (r:+0.97, f: *p*<0.001) or by quantitative immunofluorescence (r:+0.96, f: *p* = 0.02), and to the expression of antioxidant enzymes genes (r:+0.78, f: *p*<0.05; for SOD-2 expression). As expected, the TGF-β1 treatment does not induce a significant increase of ROS generation and of HIF-1α mRNA (data not shown).

Taken together, these data show that the overproduction of intracellular ROS generated from *Kp* infection at MOI 25:1 is able to determine a positively regulate transcription and synthesis of the HIF-1α.

### *K*. *pneumoniae* is able to activate EMT transcription factors in A549 epithelial cells

Many of the Enterobacteriaceae are known to trigger the initial steps of biological processes that culminate with morphological changes in cells shape and in the microenvironment, typical of an Epithelial-Mesenchimal Transition.

We wanted to test the ability of *K*. *pneumoniae* strains to induce an EMT-like process in A549 cells. To this purpose, we infected A549 cells grown in monolayers with all *Kp* strains as above. After four hours from infection, the RNAs extracted from lysed cells were subjected to real time RT-PCR to evaluate the expression of various transcription factors associated with EMT: Twist 1–2, Snail 1–2 and ZEB 1–2. A549 uninfected cells or cells treated with TGF-β1 50 ng/ml were used to control negative and positive induction of the EMT biology.

**Fig 5:**
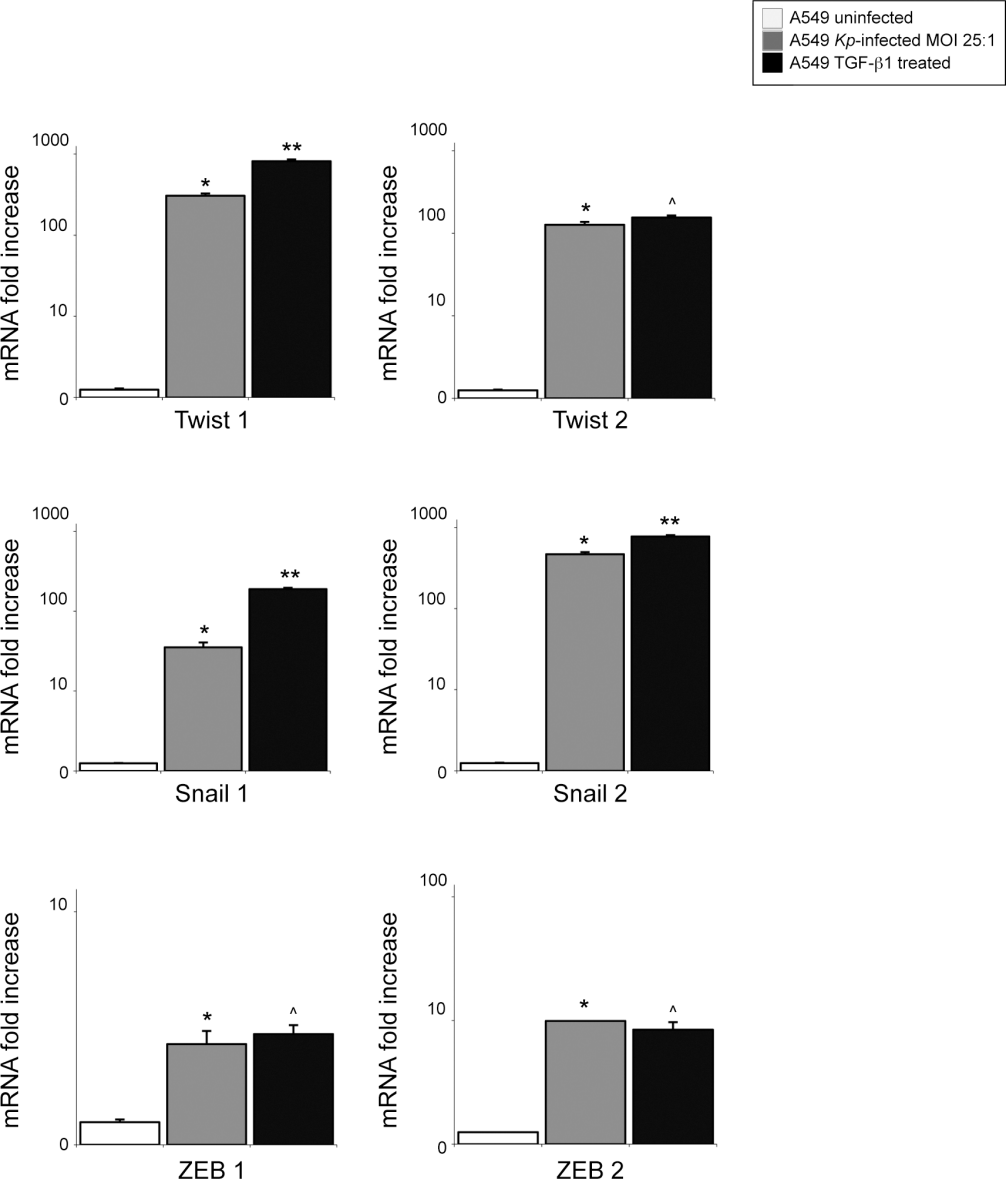
Analysis of EMT transcription factors expression in cultured airway epithelial cells infected with *K*. *pneumoniae* strains. Snail 1–2, Twist 1–2 and Zeb 1–2 mRNA levels were assessed by real-time RT-PCR in A549 *Kp*-infected cells at MOI 1:25 and normalized respect to uninfected A549 cells. A459 TGF-β1-treated cells were used as positive control of EMT-induction. Results are expressed as mean ± SD. All the *K*. *pneumoniae* strains were able to induce a significant increase of Twist 1–2, Snail 1–2 and Zeb 1–2 mRNA production (Mann-Whitney test: *p<0.01 vs uninfected). The amount of TFs in cells infected by *Kp* strains show levels of induction more similar to that obtained treating A549 cells with TGF-ß1 (Mann-Whintey test: **p<0.05 or ^*p* = not significant vs *Kp*-infected cells).

As expected, the molecular analysis confirm that cells treated with TGF-β1 respond with a generalized increase of all EMT transcription factors as compared to untreated cells (*p*<0.01; Fig 5).

All the *K*. *pneumoniae* strains were able to induce a significant increase of Twist, Snail and Zeb mRNA, showing levels of induction more similar to that obtained treating A549 cells with TGF-β1 (Fig 5)

Our results indicate that *K*. *pneumoniae* is able to trigger an EMT-like program in A549 cells by inducing the expression of the major TFs governing the transition of epithelial cells towards a mesenchymal-like phenotype.

### The scavenging of intracellular ROS counteract the expression of HIF-1α and the up-regulation of TFs involved in EMT-like process

To assess whether the intracellular ROS production could be directly responsible for the impairment of the EMT-like process triggered by *Kp* strains, the A549 cells were pretreated for 12h before the infection with resveratrol 50 μM, a natural molecule with anti-inflammatory and antioxidant action, able to exert strong inhibitory effects on ROS generation in cells stimulated with lipopolysaccharides [31–33].

**Fig 6:**
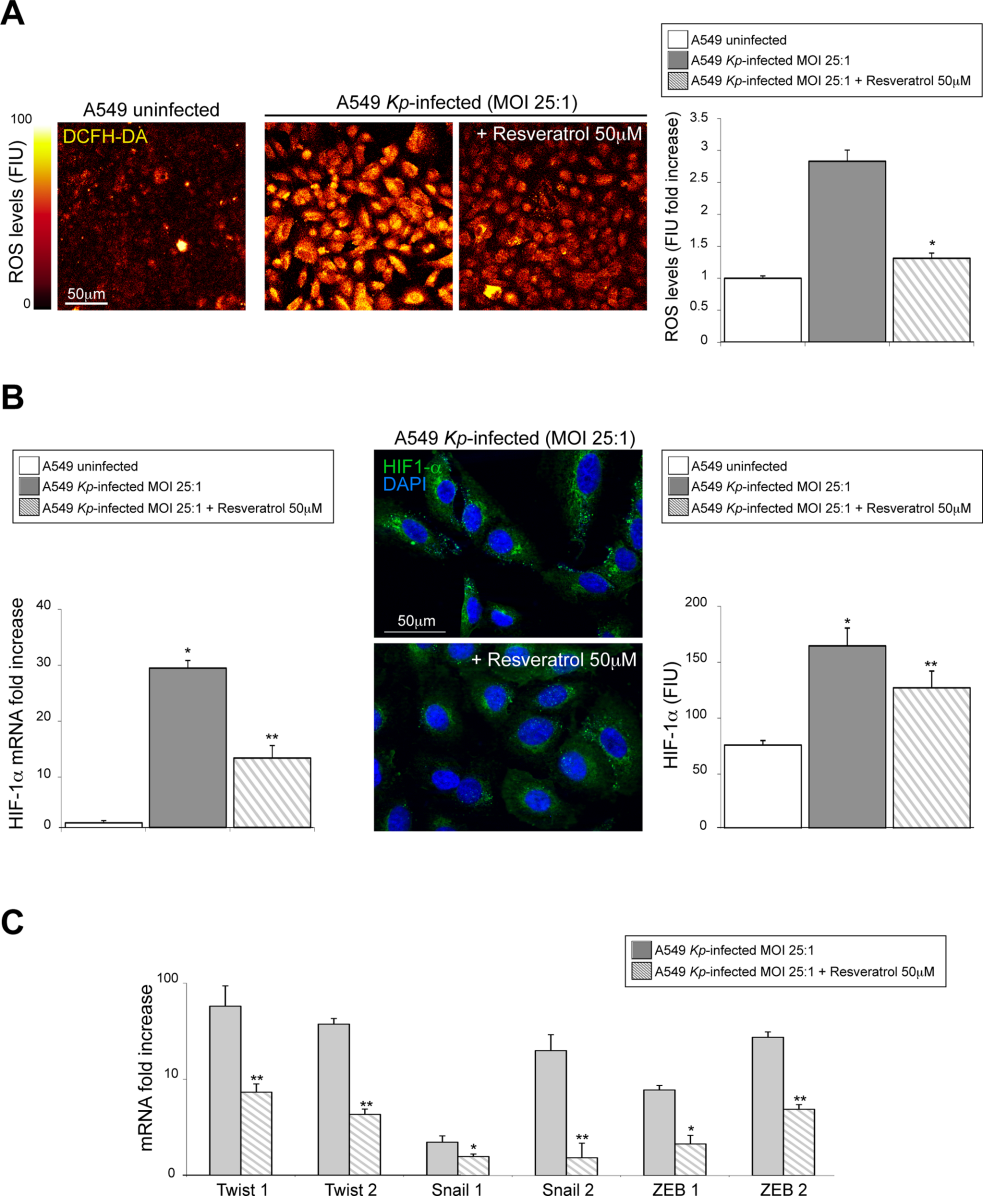
Effects of ROS scavenging on the counteraction of EMT-like process due to *K*. *pneumoniae* infection of A549 cells. The A549 cells were pretreated for 12h before the infection with the resveratrol 50 μM. (A) The pretreatment with antioxidant has been shown to significantly decrease the oxidative stress in cells infected by all strains of *K*. *pneumoniae* (Student T test: *p<0.0001 vs A549 *Kp*-infected cells without resveratrol). Legend: MFI: Mean Fluorescence Intensity; FIU: Fluorescence Intensity Units. (B) After resveratrol, has been observed a significant impairment of HIF-1α mRNA expression and of HIF-1α intracellular production in A549 infected monolayers (Student T test: *p<0.05 or **p<0.01 vs A549 *Kp*-infected cells without resveratrol). (C) The real time RT-PCR analyses show that the resveratrol was able also to counteract the up-modulation of TFs related to EMT-like process dependent to *Kp* infection. (Mann-Withney test: *p<0.05 or **p<0.01 vs A549 *Kp*-infected cells without resveratrol).

In our experimental setting, resveratrol has been shown to significantly decrease the oxidative stress (Fig 6A) and the HIF-1α expression (Fig 6B) in A549 cells infected by all strains of *Kp*. As expected, the real time RT-PCR analyses show that the resveratrol was able also to counteract the up-modulation of EMT transcription factors dependent to *Kp* infection (Fig 6C).

Taken together our results overtly indicate that the sustained scavenging of ROS impairs the activation of transcriptional pathways, driving epithelial cells through an EMT-like program.

## Discussion

Several experimental evidences have been produced in the years showing how some bacterial pathogens are able to regulate the dynamics of an EMT process by the induction of transcription factors directly involved in the activation of the EMT program in infected epithelial cells. In general, chronic bacterial infections trigger and sustain locally chronic inflammatory responses able to mediate dramatic changes of tissue remodeling, sometime resembling an EM-like transition [8]. However, in some cases, these two pathogenetic elements coexist and synergize to maintain a typical pro-inflammatory microenvironment that, over time, sustains pathological remodeling typical of EMT-like processes. This appears to be true for the fibrosis of the respiratory tract where the continue deposition of collagen and proteins of the extracellular matrix is supported by chronic microbial infections [16–18]. In this case, increased fibrogenesis is further sustained by the hyper-incretion of TGF-β, major modulator of tissue repair which, in these conditions, is able to stimulate the EMT processes driving the alveolar epithelial cells toward the acquisition of a myofibroblast-like phenotype [16,17]. Further evidences come from the work of Kondo et al. demonstrating an early induction of EMT-like program in A549 cells after the in vitro administration of flagellin purified from *Bacillus subtilis*, highly similar in its amino acid sequence to flagellin from *Legionella pneumophila*. In this case, flagellin was able to amplify the effects of TGF-β via the activation of the p38 MAP kinase-dependent pathway and the induction of the NF-kB promoter [34]. The need of specific pathway activation for the induction of EMT-like processes in course of some bacterial infection is sustained by the production of intracellular ROS and HIF-1α upregulation. To this extent, Cane et al. demonstrated that the adhesion of E. coli strains to membrane receptors of human colon cells activates a signaling cascade inducing an EMT-like behavior dependent on HIF-1α expression [9]. In addition, some other studies report that LPS from Gram-negative bacteria regulates HIF-1α expression at the transcriptional level [15,35] and that this cellular response was blocked by treatment with antioxidant [36]. While these studies indicate that the activation or modulation of specific signaling pathways contribute to the induction of specific transcriptional profiles, the mechanisms involved in the activation of the EMT program during the early phases are not fully elucidated.

We previously demonstrated that *Kp* strains have a great ability to infect and induce cytotoxic effects in several in vitro cell types with the highest virulence for carbapenem-resistant *K*. *pneumoniae* strains [23].

In this scenario, we wanted to verify whether the direct interaction of *K*. *pneumoniae* with respiratory epithelial cells would be able to trigger signaling pathways sustaining the early activation of EMT processes in course of chronic inflammatory diseases of the lower respiratory tract. Our results indicate the ability of *K*. *pneumoniae* to elicit an EMT-like process in A549 cells, at least at the lower MOIs exposure. Induction of this program appears to be related to an increased production of intracellular ROS and to the activation of HIF-1α gene [3,4].

In our model, all the *K*. *pneumoniae* strains infecting the A549 cells are able to stimulate the induction of EMT-like programs through the inductive expression of TFs typically involved in the early activation of these processes and the concomitant loss of proteins normally expressed by epithelial cells. In our simple experimental setting, the activation of epigenetic programs are driven by HIF-1 and NF-кB-dependent pathways, both exploiting oxidative stress, and both necessary to integrate the responses to different primary stimuli at the level of ROS signaling. The crucial role exercised by oxidative stress on the modulation of HIF-1 and subsequently on the activation of EMT-like process has been confirmed by the counteracting effects of resveratrol, molecule with antioxidant and anti-inflammatory actions able to exert a strong inhibitory effect also on ROS produced by inflammatory cells stimulated by LPS [31–33].

Finally, on the basis of our data, the mechanisms by which *K*. *pneumoniae* sustain an evident activation of EMT-like process can only be speculated. We demonstrated that all *Kp* strains display a greater ability to infect the epithelial cells and that the bacterial cell invasion results in the induction of a stronger oxidative stress culminating with a un-programmed cell death [23]. Potentially, a greater ability to invade the host-cells could be due to modifications of LPS structure related to the activation of additional factors encoded by the plasmid carrying carbapenemase [37] or to the activation of bacterial transcriptional regulators that are able to modulate the expression of different genes, including those associated with antimicrobial resistance [38,39].

## Conclusions

Altogether, our results suggest that the activation of fully integrated pathways in response to hypoxia and oxidative stress couples metabolic responses, chemokines and growth factor release to the activation of transcriptional programs driving cells through the acquisition of a mesenchymal-like phenotype. To our knowledge, the direct involvement of bacterial pathogens in epithelial-to-mesenchimal transition has so far been described with a few species, mostly *Escherichia coli* [9] and this is the first report that indicates that strains of *K*. *pneumoniae* may promote EMT-like processes through a direct interaction with epithelial cells without the involvement of inflammatory cells. Further research is needed to better define the signaling pathways activated during the early phases of interactions between *K*. *pneumoniae* and the host cells, and to clarify a potential role of their antibiotic resistance profile in the EMT-induction. These will certainly contribute to develop new strategies to counteract the tissue remodelling in chronic infections of the respiratory epithelium.
